# Molecular Dynamics Exploration of Selectivity of Dual Inhibitors 5M7, 65X, and 65Z toward Fatty Acid Binding Proteins 4 and 5

**DOI:** 10.3390/ijms19092496

**Published:** 2018-08-23

**Authors:** Fangfang Yan, Xinguo Liu, Shaolong Zhang, Jing Su, Qinggang Zhang, Jianzhong Chen

**Affiliations:** 1 School of Physics and Electronics, Shandong Normal University, Jinan 250358, China; yanfangfang1511@163.com (F.Y.); slzhang@sdnu.edu.cn (S.Z.); sujing817@126.com (J.S.); zhangqg@sdnu.edu.cn (Q.Z.); 2 School of Science, Shandong Jiaotong University, Jinan 250357, China

**Keywords:** molecular dynamics simulations, MM-GBSA, principal component analysis, fatty acid binding proteins (FABPs), arteriosclerosis, metabolic disease

## Abstract

Designing highly selective inhibitors of fatty acid binding proteins 4 and 5 (FABP4 and FABP5) is of importance for treatment of some diseases related with inflammation, metabolism, and tumor growth. In this study, molecular dynamics (MD) simulations combined with molecular mechanics generalized Born surface area (MM-GBSA) method were performed to probe binding selectivity of three inhibitors (5M7, 65X, and 65Z) to FABP4/FABP5 with *K*_i_ values of 0.022/0.50 μM, 0.011/0.086 μM, and 0.016/0.12 μM, respectively. The results not only suggest that all inhibitors associate more tightly with FABP4 than FABP5, but also prove that the main forces driving the selective bindings of inhibitors to FABP4 and FABP5 stem from the difference in the van der Waals interactions and polar interactions of inhibitors with two proteins. Meanwhile, a residue-based free energy decomposition method was applied to reveal molecular basis that inhibitors selectively interact with individual residues of two different proteins. The calculated results show that the binding difference of inhibitors to the residues (Phe16, Phe19), (Ala33, Gly36), (Phe57, Leu60), (Ala75, Ala78), (Arg126, Arg129), and (Tyr128, Tyr131) in (FABP4, FABP5) drive the selectivity of inhibitors toward FABP4 and FABP5. This study will provide great help for further design of effective drugs to protect against a series of metabolic diseases, arteriosclerosis, and inflammation.

## 1. Introduction

Fatty acid binding proteins (FABPs) are members of superfamily of the cytoplasmic binding proteins. FABPs play an important role in the uptake, transport, and metabolic regulation of long-chain fatty acids and are also involved in other vital processes of the organism [1,2,3,4]. Since the FABPs were first reported in 1972, there have been at least nine different subtypes confirmed in mammals, including liver FABP (L-FABP/FABP1), intestinal FABP (I-FABP/FABP2), heart FABP (H-FABP/FABP3), adipocyte FABP (A-FABP/FABP4/aP2), epidermal FABP (E-FABP/FABP5/mal1), ileal FABP (Il-FABP/FABP6), brain FABP (B-FABP/FABP7), myelin FABP (M-FABP/FABP8), and testis FABP (T-FABP/FABP9) isoforms [5]. These fatty acid binding proteins exist in different tissues in various forms. Although the residue sequences among the members of FABP family are highly different, with a range of 15–70%, they have similar three-dimensional structures, namely structural topology of two α-helices and ten β-strands [2]. Studies of cultured cells not only indicate the functions of FABPs in the import, storage and export of fatty-acid as well as metabolism of cholesterol and phospholipid, but also emphasize that FABPs can be used as a potential target for treating diseases including metabolic diseases, inflammation and atherosclerosis [6,7,8].

**Figure 1:**
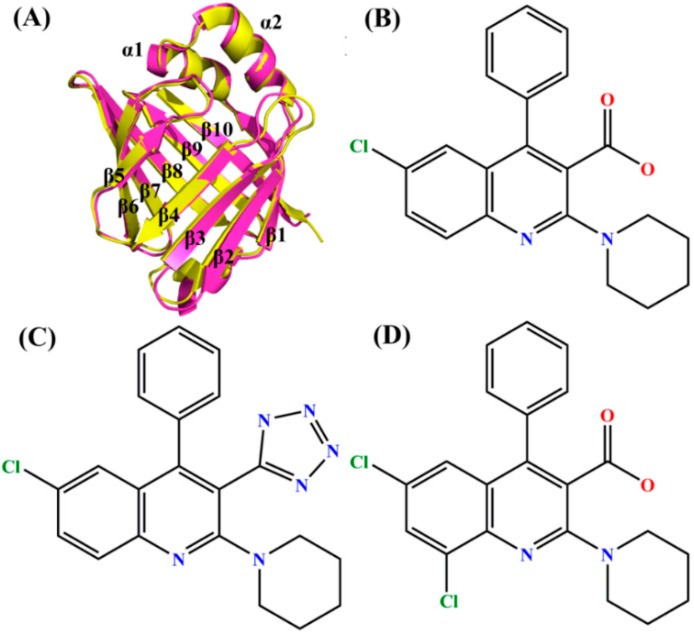
Molecular structures of fatty acid binding proteins 4 and 5 (FABP4 and FABP5), and three ligands. (**A**) Superimposed structures of FABP4 (yellow) and FABP5 (hot pink) in cartoon diagram; (**B**) 5M7; (**C**) 65X; and (**D**) 65Z.

FABP4 is first found in mature fat cells and adipose tissue [9]. The published works have shown that the expression of FABP4 is not only in macrophages, but also in the dendritic cells, which became more obvious in differentiated or activated macrophages in the monocyte cell lines of human and mouse [10,11,12,13]. Recent studies suggest that FABP4 can integrate metabolic and inflammatory responses, and plays an important role in certain aspects of metabolism, syndrome, and cardiovascular disease [14,15,16]. FABP5 is expressed most abundantly not only in endothelial and macrophages cells, but also in skin and several other tissues [13]. Both FABP4 and FABP5 are present in dendritic cells and macrophages. It is interesting to note that these two proteins have a 52% amino acid similarity and their superimposed structures are shown in Figure 1A. FABP4 and FABP5 can not only improve insulin sensitivity and the development of atherosclerosis, but also produce significant influences on certain aspects of metabolic diseases, such as diabetes and obesity [17,18,19,20,21,22].

In fact, FABP4 and FABP5 have been involved in obesity, atherosclerosis, and metabolic disease [23,24]. The protective effect of the deletion of FABP4 on atherosclerosis is related to the actions in macrophages, which has been confirmed by the bone marrow transplantation researches [25]. Moreover, the overexpression of FABP4 is also found in unstable carotid plaques [26]. Meanwhile, the overexpression of FABP5 decreases insulin sensitivity in the high-fat diet model, while the absence of FABP5 increases insulin sensitivity [27]. Compared to mice with a single deficiency of FABP4 or FABP5, the combined deficiency of FABP4 and FABP5 better improves insulin sensitivity and protects against atherosclerosis and type II diabetes [28,29]. Therefore, it is of high significance for treatment of inflammation, metabolic diseases, and inhibiting of tumor growth to develop new dual inhibitors with good pharmacokinetics and biochemical characteristics to target FABP4 and FABP5.

So far, a large number of small molecule inhibitors of FABP4 have been identified, such as atorvastatin [30], metformin [31], biphenyl azole inhibitor (BMS309403) [15], and so forth. A specific FABP4 inhibitor BMS309403 can effectively protect against diabetes mellitus, insulin resistance, and atherosclerosis in mouse model, which suggests that chemical inhibitors of FABP4 may become an effective therapeutic strategy [32]. With further understanding of biological functions of FABP family and selectivity of inhibitors toward FABP4 and FABP5, high affinity and selective dual inhibitors can be better developed and designed.

In fact, the hydrophobic ligands have high affinity and wide selectivity toward FABP4 and FABP5 [4,33]. As shown in Figure 1A, the binding pocket of FABP4 and FABP5 is located in the β-barrel. Two loops β3–β4 and β5–β6 combined with α1-loop-α2 domain form the gate to control the exit and entrance of ligands. Furthermore, there are polar and hydrophobic acids lined with the cavity in the barrel, and the carboxyl group of A-FABP can coordinate with arginine and tyrosine residues (Arg106, Arg126, and Tyr128) through electrostatic interactions [34]. To accurately clarify binding mechanisms of inhibitors to FABP4 and FABP5, three inhibitors (ID: 5M7, 65X, and 65Z) [35] were selected to probe their binding selectivity toward FABP4 and FABP5. It has been reported that the Ki values for three inhibitors 5M7, 65X, and 65Z binding to FABP4/FABP5 are 0.022/0.50 μM, 0.011/0.086 μM and 0.016/0.12 μM, respectively [35,36]. The structures of these inhibitors are depicted in Figure 1B–D. It is observed that these three inhibitors contain a common 4-phenyl quinoline scaffold. The quinoline piperidine uses a pseudo axial conformation to go into the depth of the selective pocket [36] and the piperidines usually bind to aryl rings with electron-deficient [37]. The carboxylic acid group in 5M7 (Figure 1B) was substituted by the tetrazole group to form inhibitor 65X (Figure 1C), and the inhibitor 65Z (Figure 1D) is generated by the replacement of Cl on a hydrogen atom. Understanding the differences in binding modes and binding selectivity of three inhibitors to FABP4 and FABP5 induced by different replacements from the 4-phenyl quinoline scaffold is of importance for design of dual inhibitors targeting FABP4 and FABP5.

Up until now, molecular dynamic (MD) simulations [38,39,40,41,42,43,44,45,46] and molecular mechanics generalized Born surface area (MM-GBSA) [47,48,49] method have been efficient tools to investigate binding modes of inhibitors to proteins. Furthermore, principal component (PC) analysis [50,51] has been widely applied to study the conformational changes of proteins induced by inhibitor bindings. The contributions of individual residues to binding free energies were also evaluated by using the residue-based free energy decomposition method [52]. Based on the success of the above method, we integrated these methods with dynamics analysis to probe the binding selectivity of three dual inhibitors toward FABP4 and FABP5 as well as conformational changes of two proteins induced by inhibitor bindings. We expect that this study can provide theoretical guidance for development of effective drugs to treat a series of metabolic diseases and inflammation.

## 2. Results and Discussion

### 2.1. Equilibrium and Flexibilities of Systems during Molecular Dynamics Simulations

The initial conformations used in the current simulations are shown in Figure S1. To evaluate the conformational stability of all the complex systems, root mean square deviations (RMSDs) of backbone atoms in FABP4 and FABP5 and non-hydrogen atoms in three inhibitors relative to their starting structures were calculated through 150 ns MD simulations (Figure S2). It is observed that all models tend to reach equilibrium in 60 ns of MD simulations. As shown in Figure S2B, RMSDs of all inhibitors basically reach convergence, which indicates that the structures of three inhibitors are stable during MD simulations. In addition, RMSD values of inhibitors bound to FABP4 are lower than those associated with FABP5, it can be speculated that there may be differences in the interactions of inhibitors with these two proteins and the inhibitors in FABP4 should be more stable than that in FABP5. The above results indicate that MD trajectories are reliable and can be used for further analysis.

**Figure 2:**
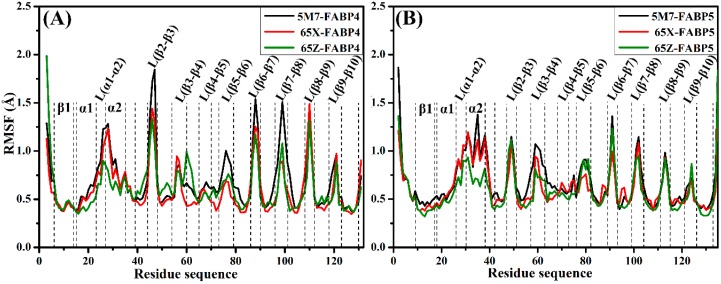
The root-mean-square fluctuations (RMSFs) of *C*_α_ atoms in FABP4 (**A**) and FABP5 (**B**).

For further understanding of the flexibility of each residue, root mean square fluctuations (RMSFs) of *C*_α_ atoms in FABP4 and FABP5 were calculated and the results were shown in Figure 2. On the whole, RMSF values of the six investigated systems have similar tendency. As seen from the RMSF values, the residues distributed near the loops show remarkable fluctuation in two proteins, additionally, the residues in α2 of FABP5 also show obvious flexibility, which implies that these residues may undergo a large conformational change due to inhibitor bindings, especially for residues Thr29 (Gly36), Asp 47 (Lys50), Phe57 (Leu60), Asp77 (Gly80), Gly89 (Gly92), Lys100 (Lys103), Asp111 (Gly114), and Val122 (Val125) in FABP4 (FABP5). Because of small fluctuation on the β-sheets, ten antiparallel β-sheets are quite rigid and stable during MD simulations, which are basically consistent with the RMSF results of other simulation analysis [53,54].

### 2.2. Differences in Internal Dynamics of FABP4 and FABP5

To better explain the differences in the internal dynamics between FABP4 and FABP5 under the same inhibitor binding, the cross-correlation coefficients between residues in the six systems were calculated and the results were plotted in Figure S3. The color-coded method was applied to describe the extent of the correlated motions between residues. According to Figure S3, the highly positive regions (red and orange) are associated with the strongly correlated movements, while the negative regions (dark and blue) represent the strongly anti-correlated motions between specific residues. Generally speaking, the regions within the range from −0.25 to 0.25 are not considered importantly.

In the case of 5M7-FABP4 and FABP5 complexes, some obvious differences between FABP4 and FABP5 are observed in the cross-correlation matrices (Figure S3A1,A2). In the 5M7-FABP4 compound (Figure S3A1), there are obviously correlated movements in the diagonal regions and the off-diagonal regions R7 and R8. The anti-correlated motions are noted in the regions R4 and R5 that respectively reflects the anti-correlated motions of β5–β6 and β7–β8 relative to the residues α1–α2. In addition, the anti-correlated motions between the residues 115–125 and 55–81 are also observed in the region R6. By comparison of Figure S3A2 with Figure S3A1, the binding of 5M7 to FABP5 extremely weakens the correlated motions of the diagonal regions in FABP5 relative to FABP4, especially for the regions of R1, R2, and R3, which are mainly distributed nearby the α1–α2, β5–β6, and β9–β10, separately. Meanwhile, the anti-correlated motions in the region R5 are also decreased by the presence of 5M7 in FABP5 compared to FABP4, and even disappear in regions R4 and R6. As shown in Figure S3B1,B2, the anti-correlated motions of the regions R4 and R5 in the 65X-FABP5 system (Figure S3B2) are slightly increased compared to the 65X-FABP4 model (Figure S3B1), and a similar phenomenon is also observed in the 65Z-FABP4 and FABP5 systems (Figure S3C1,C2). The most noticeable is that the regions R7 and R8 show strongly correlated motions relative to the residues 2–17 (5–20 in FABP5) among six systems, it can be speculated that these regions may involve strong interactions with three inhibitors. The above results suggest that regions R1–R7 may undergo larger conformational changes and these areas may be used as the potential targets of drug design.

### 2.3. Principal Component Analyses

In the current study, PC analysis was carried out to get the detailed insight into the concerted motions of FABP4 and FABP5 based on the equilibrium phase of MD simulations, and a plot of eigenvalues derived from the diagonalization of the covariance matrix against the corresponding eigenvector indices were depicted in Figure S4. It can be observed that the amplitudes of the first few eigenvalues decrease rapidly to achieve a series of constrained and more localized fluctuations. The first four principal components account for 63.6%, 56.1%, 54.5%, 51.3%, 52.8%, and 50.0% of the total motions for the 5M7-, 65X- and 65Z-FABP4 complexes and the 5M7-, 65X-, and 65Z-FABP5 complexes, respectively. It is found that the first few eigenvalues in FABP4 are higher than ones in FABP5, which indicates that the motion strength in the inhibitor-FABP4 complexes is slightly stronger than that in the inhibitor-FABP5 complexes.

**Figure 3:**
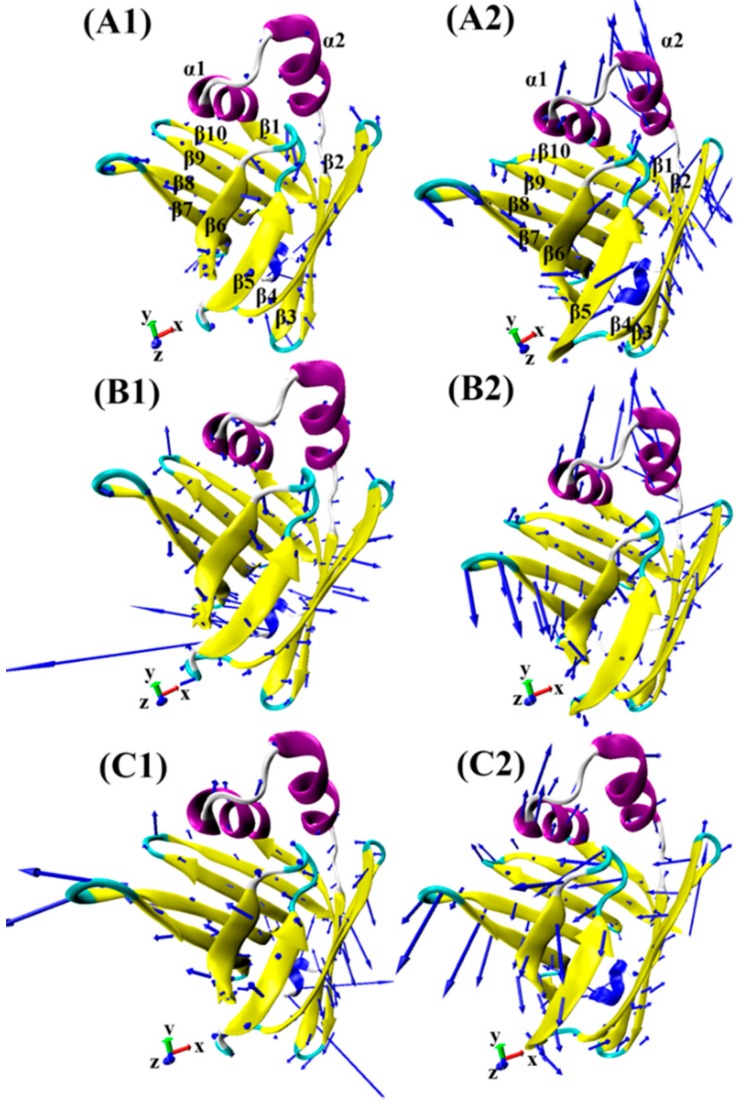
Collective motions of FABP4 and FABP5 corresponding to PC1 obtained from principle component analysis, and the direction and length of the arrows were used to reflect the direction and strength of motions. (**A1**/**A2**) 5M7-FABP4/FABP5, (**B1**/**B2**) 65X-FABP4/FABP5, and (**C1**/**C2**) 65Z-FABP4/FABP5. β-strands, loops and α-helices of FABP4/5 were displayed by using yellow, light blue and purple, respectively.

In order to qualitatively understand the difference in motional patterns between FABP4 and FABP5, six porcupine plots were generated by performing the extreme projections of MD trajectories on the first principal component PC1 (Figure 3). The direction of the arrow is indicative of the direction of motions and the length of the arrow reflects the strength of movements. According to Figure 3, the intensity of motions in FABP4 and FABP5 is significantly different, especially for the helixes α1 and α2 and the loop linking β7 with β8. In three FABP4 complexes (Figure 3A1–C1), two helixes α1 and α2 show slight movements, while these two helixes in three FABP5 models (Figure 3A2–C2) have stronger motions. Additionally, the loop linking β7 with β8 in three FABP5 complexes also shows stronger motion tendency relative to FABP4. By comparing these six investigated models, the changes in motion modes of two proteins should be attributed to the differences in the interactions of inhibitors with two proteins, which provide a hit that the binding ability of three inhibitors to FABP4 should be stronger than that of inhibitors with FABP5. Moreover, the different replacements from the 4-phenyl quinoline scaffold of three inhibitors have different influence on the direction and strength of movement for some residues in two proteins, which become more obvious for β3 and β4.

### 2.4. Binding Free Energy Analysis

To better assess the difference in the binding ability of three inhibitors to FABP4 and FABP5, the MM-GBSA method was used to calculate binding free energies of six considered systems based on 200 snapshots taken from the last 90 ns of MD trajectories with a time interval of 450 ps. Forty conformations were selected from the previous 200 snapshots at an interval of five snapshots to compute the contributions of entropy changes (−TΔS) to the inhibitor bindings by using the normal mode analysis (NMA) method. The calculated results were listed in Table 1.

As shown in Table 1, binding free energy is divided into five individual components, namely, van der Waals interaction, electrostatic interaction, polar solvation energy, nonpolar interaction, and entropic contribution. Although the calculated binding free energies ($∆G_{\mathrm{bind}}$) are higher than the experimental values ($∆G_{\exp}$), the trends of the calculated results is consistent with that of the experimental values. Furthermore, the binding free energies of inhibitors to FABP4 are stronger than that of inhibitors to FABP5, which suggests that inhibitors have stronger binding ability to FABP4 than FABP5.

To probe the origin driving the selective bindings of three inhibitors toward FABP4 and FABP5, we compared the individual components of binding free energies. It is observed that the van der Waals interactions (∆*E*_vdw_) and nonpolar interactions (∆*G*_nopol_) are beneficial to inhibitor bindings, but the term ∆*E*_vdw_ has stronger contributions to the inhibitor-protein bindings than the nonpolar term ∆*G*_nopol_. The van der Waals interactions of 5M7, 65X, and 65Z with FABP4 are decreased by 2.45, 1.26, and 1.64 kcal/mol relative to that of three inhibitors with FABP5 respectively, while the non-polar interactions (∆*G*_nopol_) of three inhibitors with two proteins do not change obviously. This result suggests that the van der Waals interactions are responsible for partial contributions to the selectivity of three inhibitors toward FABP4 and FABP5. According to Table 1, the favorable electrostatic interactions of 5M7, 65X, and 65Z with FABP4 are strengthened by 7.57, 13.83, and 13.67 kcal/mol compared to that of three inhibitors with FABP5 separately, but the unfavorable polar solvation energies (Δ*G*_pol_) of 5M7, 65X, and 65Z with FABP4 are increased by 2.08, 8.06, and 9.45 kcal/mol relative to that of three inhibitors with FABP5, respectively. Totally, because of the counteracting of the polar solvation energies on the electrostatic interactions, the unfavorable polar interactions (Δ*G*_ele+pol_) of 5M7, 65X, and 65Z with FABP4 are 5.49, 5.86, and 4.3 kcal/mol weaker than that of these three inhibitors to FABP5, respectively, which shows that the polar interactions also provide partial contributions to the selectivity of inhibitors toward FABP4 and FABP5. It is observed from Table 1 that the entropy changes, an unfavorable factor to inhibitor bindings, also contribute small forces to the selectivity of inhibitors on FABP4 and FABP5. Based on the above results, the difference in the van der Waals interactions and polar interactions of inhibitors with two proteins mostly drive the selectivity of inhibitors toward FABP4 and FABP5. With the aim to test our conclusions, we recalculate the contribution of individual component to binging free energy for five times based on 200 snapshots taken from the last 90 ns of MD trajectories with five different time intervals. The differences in the contributions of individual free energy components between FABP4 and FABP5 systems are shown in Table S1, which are basically consistent with our current analyses. In addition, the standard deviation (SD) values in Table S1 are relatively small, which indicates that our analyses and conclusions are reliable.

### 2.5. Contributions of Separated Residues to Inhibitor Bindings

**Figure 4:**
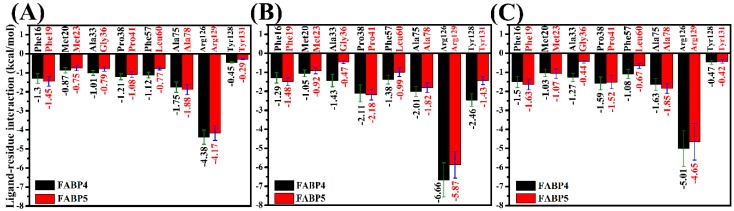
Comparisons between ligand-residue interactions of FABP4 and FABP5: (**A**) 5M7; (**B**) 65X; and (**C**) 65Z.

**Figure 5:**
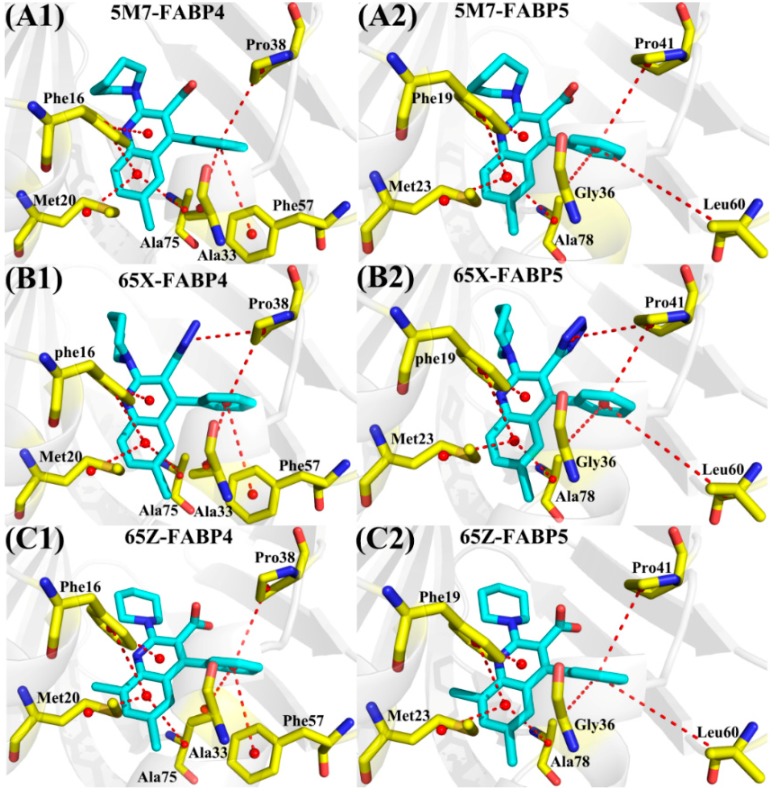
Geometric positions of inhibitors relative to the key residues involving significant interactions, the averaged distances between atoms involving significant interactions were calculated and displayed in the red lines. (**A1**/**A2**) 5M7-FABP4/FABP5; (**B1**/**B2**) 65X-FABP4/FABP5; and (**C1**/**C2**) 65Z-FABP4/FABP5. All inhibitors and key residues in inhibitor-FABP4/5 complexes are separately shown in light blue and yellow.

**Figure 6:**
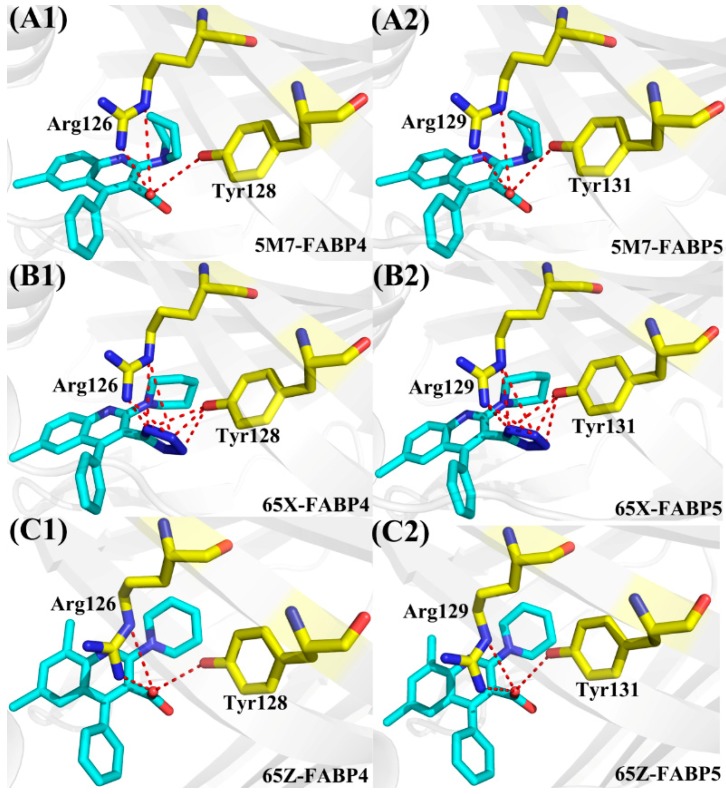
Hydrogen bonds formed between three inhibitors and FABP4/FABP5, which is displayed in red dot line. (**A1**/**A2**) 5M7-FABP4/FABP5; (**B1**/**B2**) 65X-FABP4/FABP5; and (**C1**/**C2**) 65Z-FABP4/FABP5. All inhibitors and key residues in inhibitor-FABP4/5 complexes are separately shown in light blue and yellow.

To better explain the selective mechanisms of inhibitors toward FABP4 and FABP5, the residue-based free energy decomposition method was adopted to evaluate the contributions of individual residues to binding free energies of inhibitors to FABP4 and FABP5 (Figure 4 and Figure 5). Meanwhile, the hydrogen bonding interactions of inhibitors with two proteins in the six systems were also analyzed by using the CPPTRAJ module in Amber 16, and the corresponding results are shown in Table 2 and Figure 6.

To better understand the selective mechanisms of inhibitors toward two proteins, the residues with larger contributions to the total binding free energies and the corresponding energy values were compared (Figure 4). It is noted that seven residues, including Phe16 (Phe19), Met20 (Met23), Ala33 (Gly36), Pro38 (Pro41), Phe57 (Leu60), Ala75 (Ala78), and Arg126 (Arg129), play important roles in the associations of inhibitors 5M7 and 65Z with FABP4 (FABP5) and eight key residues, including Phe16 (Phe19), Met20 (Met23), Ala33 (Gly36), Pro38 (Pro41), Phe57 (Leu60), Ala75 (Ala78), Arg126 (Arg129), and Tyr128 (Tyr131), provide key contribution to the binding of 65X to FABP4 (FABP5). The geometrical positions of key residues relative to inhibitors are shown in Figure 5 and Figure 6. Considering that the substitution of residues in the binding sites may have an impact on the selective binding of inhibitors toward FABP4/5, the energy contribution of substituted residues in or near the binding sites of FABP4 and FABP5 was analyzed (Table S2). As shown in Table S2, residues Ala33 (Gly36) and Phe57 (Leu60) in FABP4 (FABP5) have significant contribution not only to the binding free energy, but also to the selective binding compared to the other substituted residues. According to Figure 5, structurally the phenyl ring of Phe16 (Phe19 in FABP5) can form the π-π stack interactions with the diphenyl groups of three inhibitors. Moreover, totally the π-π stack interactions of the residue Phe16 in FABP4 with three inhibitors are weaker than that of Phe19 in FABP5 with inhibitors (Figure 4). The alkyls of the residues Met20 in FABP4 and Met23 in FABP5 can form the CH-π interactions with the phenyl groups of inhibitors and the interaction intensity does not exist obvious difference between two proteins. Pro38 in FABP4 and Pro41 in FABP5 can also form the π-π stack interaction with the phenyl ring of three inhibitors. It is worth noting that the interactions of Pro38 in FABP4 and Pro41 in FABP5 with 65X, which respectively corresponding to −2.11 and −2.18 kcal/mol, are stronger than that of Pro38 in FABP4 and Pro41 in FABP5 with 5M7 and 65Z. The cause leading to this result is that these residues produce an additional interaction with the tetrazole of 65X. In three FABP4 complexes, the alkyls of Ala33 are close to the phenyl groups of 5M7, 65X, and 65Z, which respectively form the CH-π interactions, while the carbon atoms of Gly36 can also separately generate the CH-π interactions with inhibitors in three FABP5 complexes (Figure 5). It is observed that the interactions of 5M7, 65X, and 65Z with Ala33 in FABP4 are increased by 0.22, 0.96, and 0.83 kcal/mol compared to that of three inhibitors with Gly36 in FABP5, respectively, which is owed to the length of the side-chain of Gly36 shorter than that of Ala33. The alkyls of the residues Ala75 and Ala78, respectively corresponding to FABP4 and FABP5, are close to the phenyl groups of three inhibitors and produce the CH-π interactions with three inhibitors in six systems to maintain the structural stability (Figure 5). According to Figure 4, the interactions of 5M7 and 65Z with Ala75 in FABP4 are decreased by 0.13 and 0.22 kcal/mol compared to that Ala78 in FABP5 separately, but the interaction of Ala75 with 65X is increased by 0.19 kcal/mol relative to that of Ala78 with 65X. According to Figure 5A1, B1, and C1, the phenyl of Phe57 in FABP4 is located near the phenyl groups of 5M7, 65X, and 65Z, which separately forms the π-π stack interactions between Phe57 and three inhibitors. Structurally, the alkyl of Leu60 in FABP5 can produce the CH-π interactions with the phenyl rings of all studied inhibitors. It is found that the interactions of Phe57 with 5M7, 65X, and 65Z are strengthened by 0.35, 0.39, and 0.41 kcal/mol relative to that of Leu60 with 5M7, 65X, and 65Z, which suggests that the π-π stack interactions between Phe57 and inhibitors are stronger than CH-π interactions of Leu60 with inhibitors.

As shown in Figure 4, the residues Arg126 in FABP4 and Arg129 in FABP5 produce the strongest interactions with three inhibitors. To reveal the cause leading to this result, hydrogen bonding interactions and polar interactions were analyzed by using CPPTRAJ module and residue-based free energy decomposition method. According to Table 2, the carboxylic acid groups of inhibitors 5M7 and 65Z, respectively, form two hydrogen bonds with Arg126 in FABP4 and Arg129 in FABP5 (Figure 6A1/A2,C1/C2). More importantly, the negative charge of the carboxylic acid group in 5M7 and 65Z generates strong electrostatic interactions with the positive charge of Arg126 and Arg129. For the inhibitor 65X, the replacement of the tetrazole group in 65X on the carboxylic acid group in 5M7 and 65Z not only produces four hydrogen bonds with 65X, but also strong electrostatic interaction between the negative charge of the tetrazole group and the positive charge of Arg126 and Arg129. Thus, the electrostatic interactions and hydrogen bonds are the main cause leading to the strongest interactions of Arg126 and Arg129 with three inhibitors among all the residues. It’s worth mentioning that the interactions of Arg126 in FABP4 with 57M, 65X, and 65Z are increased by 0.21, 0.79, and 0.36 kcal/mol relative to that of Arg129 in FABP5 with these inhibitors, separately. In addition, the carboxylic acid group in 5M7 and 65Z also form a hydrogen bond with Tyr128 in FABP4 and Tyr131 in FABP5, while the tetrazole group of 65X produces three hydrogen bonding interactions with Tyr128 in FABP4 and Tyr131 in FABP5 (Table 2 and Figure 6). This result reasonably explain the cause why the interactions of Tyr128 and Tyr31 with 65X are stronger than that of these two residues with 5M7 and 65Z. Except for the hydrogen bonds Arg126-NH2-HH21···65Z-O21 and Arg129-NH2-HH21···65Z-O21, the occupancy of all hydrogen bonds between inhibitors and FABP5 is decreased compared to that between FABP4 and inhibitors.

**Figure 7:**
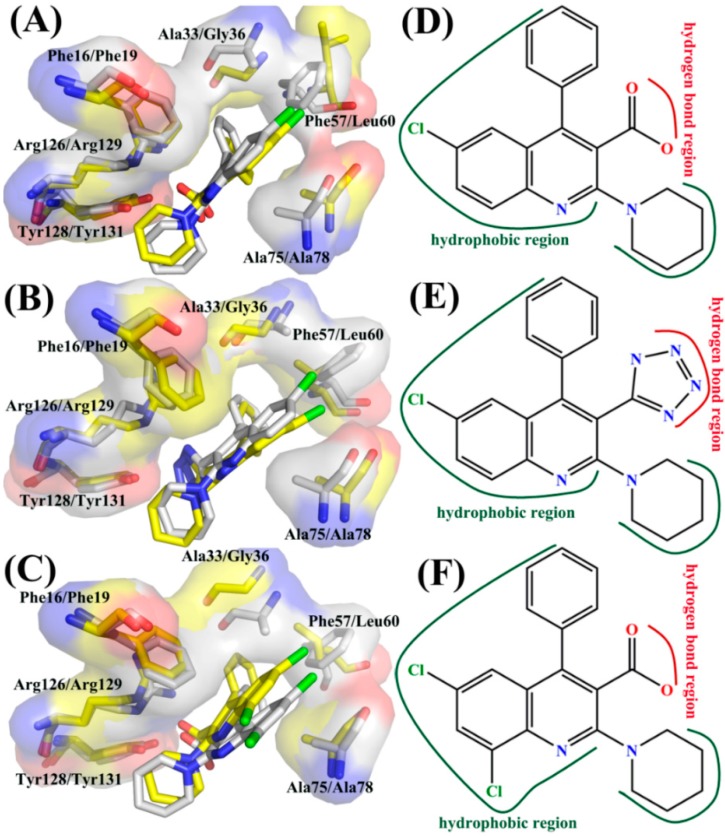
Superposition of specific active sites between structures of inhibitor-FABP4 complex and inhibitor-FABP5 complex: (**A**) 5M7-FABP4/FABP5; (**B**) 65X-FABP4/FABP5; and (**C**) 65Z-FABP4/FABP5. Inhibitors and key residues in inhibitor-FABP4 complexes are displayed with gray, while that in inhibitor-FABP5 complexes are displayed with yellow. The interaction models between FABP4/5 and three inhibitors: (**D**) 5M7; (**E**) 65X; and (**F**) 65Z.

Based on the above analyses, three inhibitors 5M7, 65X, and 65Z show selectivity toward FABP4 against FABP5. It is observed that the main force driving the selectivity of inhibitors on two proteins come from the residues (Phe16, Phe19), (Ala33, Gly36), (Phe57, Leu60), (Ala75, Ala78), and (Arg126, Arg129) separately corresponding to (FABP4, FABP5). In addition, the difference in the interaction of Tyr128 in FABP4 and Tyr131 in FABP5 with 65X also provides significant contribution to the selectivity of 65X toward two proteins. The binding difference of three inhibitors to residues (Phe16, Phe19), (Ala33, Gly36), (Phe57, Leu60), and (Ala75, Ala78) in (FABP4, FABP5) contributes the hydrophobic driving force to the selectivity of three inhibitors toward FABP4 and FABP5, while the difference in the interactions of inhibitors with (Arg126, Arg129) in (FABP4, FABP5) provide polar driving force for the selective bindings of three inhibitors to FABP4 and FABP5. For the 65X-FABP4/FABP5 complexes, the difference in the interaction of Tyr128 and Tyr131 with 65X also contribute partially force to the selectivity of this inhibitor. Thus, designs of potent dual inhibitors targeting FABP4 and FABP5 should give full consideration to the binding difference of inhibitors to the key residues in FABP4 and FABP5. In order to provide better theoretical guidance for the design of new inhibitors, we summarized the binding modes of three inhibitors to some key residues. The theoretical pharmacophore models [55] of inhibitors for FABP4/5 and the specific active sites for structures of inhibitor-FABP4 and inhibitor-FABP5 complexes are shown in Figure 7.

## 3. Materials and Methods

### 3.1. Molecular Docking

AutoDock4.2 [56] software package was applied to achieve molecular docking of inhibitors with FABP4 and FABP5. The crystal structures of 5M7-FABP4 complex (PDB ID: 5EDC [36]) and 65X-FABP5 compound (PDB ID: 5HZ5 [35]) obtained from Protein Data Bank (PDB) were used as templates for docking studies, and three inhibitors 5M7, 65X, 65Y were docked into the binding pockets of PABP4 and FABP5 to produce the missing inhibitor-protein complexes. All the water, buffer molecules and ions were discarded [57]. The missing hydrogen atoms were added and the Gasteiger charges were calculated in the process of preparing inhibitors and proteins. The inhibitor-protein docking was performed by using the Lamarckian genetic algorithm (LGA). During the docking, the grid box for docking studies was set to (60, 60, 60) in (*x*, *y*, *z*) direction with a spacing value of 0.375 Å. The parameters were set to the default values of the software. Finally, the best conformations were chosen based on the predicted binding free energy for the following MD simulations.

### 3.2. Molecular Dynamics Simulations

Initializations and MD simulations of all complexes were carried out by using the Amber 16 [58] software. The structures of three inhibitors were optimized at the semiempirical AM1 level, and the atomic charges were assigned to inhibitors by applying the AM1-BCC program [59,60] implemented in Amber 16. The force field parameters of inhibitors and proteins were generated by the general Amber force field (GAFF) and Amber FF99SB [61] force field, respectively. Then, all the systems were solvated in a truncated octahedral box of TIP3P water molecules with a separation margin of 12.0 Å from the solute along each dimension [62,63]. An appropriate number of counterions were placed to maintain neutral charge of systems.

To remove any unfavorable factors formed by the initialization of systems, each system was subject to two-stage energy minimizations before starting MD simulations. Firstly, water molecules and ions were minimized by restraining the complex with a harmonic constant of 100 kcal·mol^−1^·Å^−2^. Secondly, the whole system is optimized without any restrictions. Moreover, the combination of the steepest descent and conjugated gradient methods were performed in each stage. After that, the systems endure a slowly heating process from 0 K to 300 K in 1 ns and then equilibrated for another 1 ns at constant pressure of 1 atm and normal temperature of 300 K. Finally, 150-ns MD simulations without any restrictions were conducted, and the conformations of each system were recorded every 4 ps. The Sander module in Amber 16 was adopted to perform all current simulations. During the simulations, the SHAKE algorithm is used to restrain the chemical bonds involving hydrogen atoms [64], and the time step of dynamic simulations is set as 2 fs. The Langevin thermostat with a collision frequency of 2.0 ps^−1^ was used to regulate the temperature of each system. The long-range electrostatic interactions were calculated by employing the particle mesh Ewald (PME) method [65,66]. The electrostatic and van der Waals interactions were truncated at a suitable distance of 9.0 Å. The CPPTRAJ [67] program in Amber 16, PyMOL (http://www.pymol.org.) and VMD [68] programs were adopted to perform analyses on MD results and depict pictures.

### 3.3. Principal Component Analysis

In order to probe internal dynamics of proteins, the cross-correlation analysis was performed by calculating the cross-correlation matrix C using the coordinates of $C_{\alpha }$ atoms from the equilibrium MD trajectories. The matrix C can reasonably evaluate the fluctuations of $C_{\alpha }$ atoms relative to its averaged positions and the cross-correlation coefficient $C_{\mathrm{ij}}$ can be computed by the flowing equation [69]:

(1) $$C_{\mathrm{ij}}=\frac{\langle ∆r_{i}∆r_{j}\rangle }{{\left(\langle ∆r_{i}^{2}\rangle \langle ∆r_{j}^{2}\rangle \right)}^{1/2}}$$

in which the angle bracket indicates the time average over the equilibrated trajectories of MD simulations, and $∆r_{i}$ is the displacement from the averaged position of the ith atom. The range of $C_{\mathrm{ij}}$ values is from −1 to 1. The positive value of $C_{\mathrm{ij}}$ depicts the correlated motion between residues i and j, while the negative value of $C_{\mathrm{ij}}$ describes the anti-correlated motion of residue i relative to j. In addition, PC analysis was also carried out to further study the conformational changes of proteins by constructing a covariance matrix C of atomic coordinates from MD trajectories, which can be used to identify the essential degrees of freedom in the movements of proteins. The elements of the matrix C were given by the flowing equation:

(2) $$C_{\mathrm{ij}}=\langle (r_{i}-\langle r_{i}\rangle ){\left(r_{j}-\langle r_{j}\rangle \right)}^{T}\rangle \left(i,j=1,2,3,\ldots ,3N\right)$$

where $r_{i}$ is the Cartesian coordinates of $C_{\alpha }$ atoms in the ith residue and the average is computed based on the equilibrated phase of trajectories. *N* is the number of $C_{\alpha }$ atoms involved in current calculations. The least-square fit procedure was adopted to superpose protein on a reference structure to eliminate overall translations and rotations [70,71]. An orthogonal coordinate transformation matrix T can be employed to transform the symmetric matrix C into a diagonal matrix Ʌ of eigenvalues $\lambda_{i}:$

(3) $$\Lambda =T^{T}C_{\mathrm{ij}}T$$

in which the columns are the eigenvectors describing the directions of motions relative to $\langle r_{i}\rangle$, and the eigenvalues represent the amplitudes of motions along the corresponding eigenvectors. Meanwhile, the significant motions of proteins can be represented by the first few principal components. In the current study, the CPPTRAJ module in Amber 16 was applied to perform PC and cross-correlation analyses.

### 3.4. MM-GBSA Calculations

By now, MM-GBSA method has been applied to successfully calculate the binding affinities of inhibitors to different targeting proteins [72,73,74,75,76,77,78,79]. Moreover, Hou’s group compared the performance of MM-GBSA and molecular mechanics Poisson Boltzmann surface area (MM-PBSA) by using different biology system [80,81,82,83,84], and their results suggest that both MM-PBSA and MM-GBSA algorithms have their own advantages and disadvantages for different biosystems, and the later shows slightly better performance in the rank of binding ability of inhibitors to proteins. Thus, binding free energy of each system was computed using MM-GBSA based on 200 conformations taken from the equilibrated MD trajectories. Moreover, water molecules and counterions were removed from the snapshots. Binding free energies (∆G) can be obtained based on the following equation:

(4) $$∆G=∆E_{\mathrm{ele}}+∆E_{\mathrm{vdw}}+∆G_{\mathrm{pol}}+∆G_{\mathrm{nopol}}-T∆S$$

where the first two terms $(∆E_{\mathrm{ele}},∆E_{\mathrm{vdw}}$) represent the electrostatic and van der Waals interactions in the gas phase, respectively, and these two terms can be calculated using FF99SB force field. The third term is the polar contribution to salvation free energies. The ionic strength, dielectric constants of proteins and solvent were set to 0.15 M, 1.0 and 80.0, respectively. The fourth term is determined by the following empirical relationship:

(5) $$∆G_{\mathrm{nonpol}}=\gamma \times \mathrm{SASA}+\beta$$

where SASA denotes the solvent accessible surface area. The values for empirical parameters γ and β were set to 0.0072 kcal·mol^−1^·Å^−2^ and 0.0 kcal·mol^−1^ in this work, respectively [85,86]. The last term −T∆S is the contribution of entropy change to binding affinity caused by the changes of motion freedom due to inhibitor bindings, which can be computed by normal mode analysis [87]. Considering that the calculation of entropy is very time-consuming, thus, only 40 conformations were selected from the 200 snapshots for the calculation of the entropy.

## 4. Conclusions

In the current work, 150 ns MD simulations were performed on six systems to investigate the selective binding of three dual inhibitors 5M7, 65X, and 65Z to FABP4 and FABP5. After 60 ns of MD simulations, all systems basically reach the equilibrium. PC analyses were carried out to probe the difference in internal dynamics between FABP4 and FABP5 caused by inhibitors binding. The results show that the inhibitors-FABP4 systems are more stable than the inhibitors-FABP5 complexes. MM-GBSA method coupled with the residue-based free energy decomposition method were performed to evaluate the binding ability of three inhibitors to FABP4 and FABP5 as well as the contributions of individual residues to binding free energies. The calculated results suggest that van der Waals interactions play an important role in the bindings of inhibitors to two proteins. Three inhibitors 5M7, 65X, and 65Z display obvious selectivity toward FABP4 over FABP5, which are mainly driven by the van der Waals interactions and polar interactions of inhibitors with these two proteins. Meanwhile, it is found that the binding difference of inhibitors to residues (Phe16, Phe19), (Ala33, Gly36), (Phe57, Leu60), (Ala75, Ala78), (Arg126, Arg129), and (Tyr128, Tyr131) in (FABP4, FABP5) drive the selectivity of three inhibitors toward FABP4 and FABP5. The hydrophobic interactions of three inhibitors with the residues (Phe16, Phe19), (Ala33, Gly36), (Phe57, Leu60), and (Ala75, Ala78) in (FABP4, FABP5) provide the main driving force for the selectivity of three inhibitors toward FABP4 and FABP5, and the selective binding is also contributed by the polar interaction of (Arg126, Arg129) in (FABP4, FABP5) with inhibitors. It is worth noting that the binding difference of (Tyr128, Tyr131) in (FABP4, FABP5) with 65x also generate partially force to the selectivity of 65x. Thus, rational optimization of these driving forces for the selective bindings of inhibitors to FABP4 and FABP5 is critical to the design of dual drugs. We expect that this work can provide theoretical helps for rational designs of effective drugs to treat a series of metabolic diseases, arteriosclerosis, and inflammation.

## Figures and Tables

**Table 1:** Binding free energies of inhibitors to FABP4 and FABP5 calculated by molecular mechanics generalized Born surface area (MM-GBSA) method ^a^.

Energy	5M7-FABP4	5M7-FABP5	65X-FABP4	65X-FABP5	65Z-FABP4	65Z-FABP5
$∆E_{\mathrm{ele}}$	−104.54 ± 7.18	−96.97 ± 6.79	−114.18 ± 4.99	−100.35 ± 8.13	−112.32 ± 4.96	−98.56 ± 7.75
$∆E_{\mathrm{vdw}}$	−42.24 ± 2.35	−44.69 ± 2.36	−44.33 ± 2.52	−45.59 ± 3.17	−42.67 ± 2.45	−44.31 ± 2.56
$∆G_{\mathrm{pol}}$	115.18 ± 3.84	113.10 ± 6.26	124.30 ± 4.31	116.24 ± 5.79	122.76 ± 3.77	113.31 ± 4.71
$∆G_{\mathrm{nopol}}$	−6.07 ± 0.16	−6.11 ± 0.13	−6.36 ± 0.12	−6.41 ± 0.13	−6.02 ± 0.14	−6.33 ± 0.13
^b^ $∆G_{\mathrm{ele}+\mathrm{pol}}$	10.64 ± 2.96	16.13 ± 2.35	10.12 ± 2.10	15.98 ± 2.11	10.44 ± 2.72	14.74 ± 2.14
−T∆S	23.75 ± 4.68	25.21 ± 5.96	24.96 ± 6.95	23.15 ± 5.33	23.91 ± 5.23	23.74 ± 6.13
^c^ $∆G_{\mathrm{bind}}$	−13.92	−9.46	−15.61	−12.96	−14.35	−12.16
^d^ $∆G_{\exp}$	−10.52	−8.66	−10.94	−9.71	−10.71	−9.50

^a^ All values are in kcal/mol. ^b^ $∆G_{\mathrm{ele}+\mathrm{pol}}=∆E_{\mathrm{ele}}+∆G_{\mathrm{pol}}$. ^c^ $∆G_{\mathrm{bind}}=∆E_{\mathrm{ele}}+∆E_{\mathrm{vdw}}+∆G_{\mathrm{pol}}+∆G_{\mathrm{nonpol}}-T∆S$. ^d^ The experimental values were derived from the experimental *K*_i_ values using the equation $∆G_{\exp}=-{\mathrm{TRlnK}}_{i}$.

**Table 2:** Main hydrogen bonding interactions formed between inhibitors and FABP4 and FABP5.

Compound	^a^ Hydrogen Bonds	Distance (Å)	Angle (°)	^b^ Occupancy (%)
FABP4-5M7	Arg126-NH2-HH21···5M7-O18	2.8	157.6	99.8
	Arg126-NE-HE···5M7-O18	3.1	137.8	79.4
	Tyr128-OH-HH···5M7-O18	3.2	128.1	4.5
FABP5-5M7	Arg129-NH2-HH21···5M7-O18	2.8	154.4	95.9
	Arg129-NE-HE···5M7-O18	3.0	141.5	77.1
	Tyr131-OH-HH···5M7-O18	3.3	128.3	2.3
FABP4-65X	Arg126-NH2-HH21···65X-N28	2.9	153.4	99.7
	Arg126-NE-HE···65X-N28	3.1	138.2	65.4
	Arg126-NH2-HH21···65X-N27	3.3	158.1	53.9
	Arg126-NE-HE···65X-N27	3.3	150.0	34.1
	Tyr128-OH-HH···65X-N26	3.2	155.5	46.4
	Tyr128-OH-HH···65X-N27	2.9	158.2	98.0
	Tyr128-OH-HH···65X-N28	3.2	138.8	57.1
FABP5-65X	Arg129-NH2-HH21···65X-N28	2.9	151.3	81.4
	Arg129-NE-HE···65X-N28	3.1	140.7	61.0
	Arg129-NH2-HH21···65X-N27	3.3	154.9	50.0
	Arg129-NE-HE···65X-N27	3.3	153.6	30.0
	Tyr131-OH-HH···65X-N26	3.1	151.3	47.9
	Tyr131-OH-HH···65X-N27	2.9	152.8	80.5
	Tyr131-OH-HH···65X-N28	3.3	137.3	25.4
FABP4-65Z	Arg126-NH2-HH21···65Z-O21	2.8	157.7	99.9
	Arg126-NE-HE···65Z-O21	3.1	136.5	89.2
	Tyr128-OH-HH···65Z-O21	3.2	129.6	6.1
FABP5-65Z	Arg129-NH2-HH21···65Z-O21	2.7	156.6	99.9
	Arg129-NE-HE···65Z-O21	3.1	138.4	82.7
	Tyr131-OH-HH···65Z-O21	3.1	130.1	5.1

^a^ Hydrogen bonds are determined by the acceptor···donor distance of <3.5 Å and acceptor···H-donor angle of >120°. ^b^ Occupancy (%) is defined as the percentage of simulation time that a specific hydrogen bond exists.

## Supplementary Materials

The following are available online at http://www.mdpi.com/1422-0067/19/9/2496/s1, Table S1. Comparison of energy contribution of individual component in inhibitor-FABP4 and inhibitor-FABP5 systems by MM-GBSA method ^a^; Table S2. Energy contribution of substituted residues in FABP4 and FABP5 calculated by MM-GBSA method ^a^; Figure S1. Superposition of conformations used in the experimental studies between inhibitor-FABP4 (yellow) and inhibitor-FABP5 (pink) complexes. (A) 5M7-FABP4/FABP5, (B) 65X-FABP4/FABP5, and (C) 65Z-FABP4/FABP5; Figure S2. Root-mean-square-deviations (RMSDs) of the backbone atoms in FABP4/FABP5 (A) and three inhibitors (B) relative to their starting structures during the MD simulation as function as time. Figure S3. Cross-correlation matrices of fluctuations of Cα atoms relative to their average positions in six systems: (A1/A2) 5M7-FABP4/FABP5, (B1/B2) 65X-FABP4/FABP5, and (C1/C2) 65Z-FABP4/FABP5; Figure S4. Comparison of the eigenvalues plotted against the corresponding eigenvector indices originated from the diagonalization of covariance matrix of Cα atoms.

## Abbreviations

FABP4 and FABP5	Fatty acid binding proteins 4 and 5
MD	Molecular dynamics
MM-GBSA	Molecular mechanics generalized Born surface area
MM-PBSA	Molecular mechanics Poisson Boltzmann surface area
FABPs	Fatty acid binding proteins
L-FABP/FABP1	Liver FABP
I-FABP/FABP2	Intestinal FABP
H-FABP/FABP3	Heart FABP
A-FABP/FABP4/aP2	Adipocyte FABP
E-FABP/FABP5/mal1	Epidermal FABP
Il-FABP/FABP6	Ileal FABP
B-FABP/FABP7	Brain FABP
M-FABP/FABP8	Myelin FABP
T-FABP/FABP9	Testis FABP
LGA	Lamarckian genetic algorithm
GAFF	General Amber force field
PME	Particle mesh Ewald
RMSDs	Root mean square deviations
RMSFs	Root mean square fluctuations
