# Supplementary material for: Molecular Dynamics Exploration of Selectivity of Dual Inhibitors 5M7, 65X, and 65Z toward Fatty Acid Binding Proteins 4 and 5
Source: Int J Mol Sci. 2018 Aug 23;19(9):2496. doi: 10.3390/ijms19092496 (PMC6164837; doi:10.3390/ijms19092496)
Supplement: Supplementary file 1 [file ijms-19-02496-s001.pdf]

# Supporting information for

# Molecular Dynamics Exploration of Selectivity of Dual Inhibitors 5M7, 65X and 65Z toward Fatty Acid Binding Proteins 4 and 5

**Fangfang Yan[1], Xinguo Liu[1,*], Shaolong Zhang[1], Jing Su[1], Qinggang Zhang[1] and Jianzhong Chen[2,*]**

[1] School of Physics and Electronics, Shandong Normal University, Jinan, 250358, China;

[2] School of Science, Shandong Jiaotong University, Jinan, 250357, China

[*] Corresponding: liuxinguo@sdnu.edu.cn (X. Liu); chenjianzhong1970@163.com and jzchen@sdjtu.edu.cn (J. Chen)

Table S1. Comparison of energy contribution of individual component in inhibitor-FABP4 and inhibitor-FABP5 systems by MM-GBSA method[a]

| Energy | $\Delta\Delta E_{ele}$ | $\Delta\Delta E_{vdw}$ | $\Delta\Delta G_{pol}$ | $\Delta\Delta G_{nopol}$ | $^{b}\Delta\Delta G_{ele+pol}$ |
|---|---|---|---|---|---|
| 5M7-FABP4/5 | -7.30 | 3.06 | 1.19 | 0.03 | -6.12 |
| | -6.84 | 3.02 | 1.46 | 0.04 | -5.27 |
| | -7.35 | 3.45 | 1.22 | 0.01 | -6.03 |
| | -7.63 | 3.08 | 1.54 | 0.04 | -6.09 |
| | -7.66 | 3.14 | 1.97 | 0.07 | -5.69 |
| AVE | -7.36 | 3.15 | 1.48 | 0.04 | -5.84 |
| SD | 0.30 | 0.15 | 0.28 | 0.02 | 0.32 |
| 65X-FABP4/5 | -14.26 | 1.95 | 8.66 | 0.04 | -5.60 |
| | -14.01 | 1.64 | 8.80 | 0.04 | -5.21 |
| | -13.10 | 1.85 | 7.02 | 0.03 | -6.08 |
| | -14.24 | 1.45 | 8.22 | 0.07 | -6.02 |
| | -14.08 | 1.35 | 8.30 | 0.06 | -5.77 |
| AVE | -13.94 | 1.68 | 8.2 | 0.05 | -5.74 |
| SD | 0.43 | 0.23 | 0.63 | 0.01 | 0.31 |
| 65Z-FABP4/5 | -13.33 | 1.68 | 8.65 | 0.29 | -4.68 |
| | -14.12 | 1.44 | 9.47 | 0.33 | -4.65 |
| | -14.61 | 1.52 | 9.10 | 0.31 | -5.51 |
| | -12.84 | 2.05 | 8.45 | 0.32 | -4.39 |
| | -14.21 | 1.70 | 9.51 | 0.31 | -4.70 |
| AVE | -13.82 | 1.68 | 9.04 | 0.31 | -4.79 |
| SD | 0.64 | 0.21 | 0.43 | 0.01 | 0.38 |

[a]All values are in kcal/mol.

$\Delta\Delta E=\Delta E^{FABP4}-\Delta E^{FABP5}$

$$SD=\sqrt{\frac{1}{5}\sum_{i=1}^{5}\left(\Delta\Delta E^{i}-\Delta\Delta E^{ave}\right)^{2}}$$

Table S2. Energy contribution of substituted residues in FABP4 and FABP5 calculated by MM-GBSA method[a].

| | 5M7-FABP4/5 | | [b]ΔΔE | 65X-FABP4/5 | | [b]ΔΔE | 65Z-FABP4/5 | | [b]ΔΔE |
|---|---|---|---|---|---|---|---|---|---|
| Ser12/Asp15 | 0.02 | 0.16 | -0.14 | 0.02 | 0.18 | -0.16 | 0.02 | 0.17 | -0.15 |
| Glu14/Lys17 | 0.18 | 0.17 | 0.01 | -0.18 | -0.09 | -0.09 | 0.18 | 0.09 | 0.09 |
| Asp18/Glu21 | 0.11 | 0.11 | 0 | 0.12 | 0.12 | 0 | 0.11 | 0.12 | -0.01 |
| Val23/Leu26 | -0.34 | -0.3 | -0.04 | -0.51 | -0.38 | -0.13 | -0.52 | -0.35 | -0.17 |
| Phe27/Ile30 | -0.01 | -0.01 | 0 | -0.01 | -0.01 | 0 | -0.01 | -0.01 | 0 |
| Thr29/Leu32 | -0.43 | -0.47 | 0.04 | -0.32 | -0.43 | 0.11 | -0.34 | -0.45 | 0.11 |
| Val32/Met35 | -0.23 | -0.41 | 0.18 | -0.16 | -0.27 | 0.11 | -0.29 | -0.22 | -0.07 |
| Ala33/Gly36 | -1.01 | 0.79 | -0.22 | -1.43 | -0.47 | -0.96 | -1.27 | -0.44 | -0.82 |
| Gly34/Ala37 | 0.01 | -0.05 | 0.06 | -0.02 | -0.04 | 0.02 | 0 | -0.03 | 0.03 |
| Asn39/Asp42 | -0.01 | 0.17 | -0.18 | -0.05 | -0.22 | 0.17 | -0.01 | -0.2 | 0.19 |
| Met40/Cys43 | -0.13 | -0.09 | -0.04 | -0.1 | -0.07 | -0.03 | -0.1 | -0.07 | -0.03 |
| Ser53/Thr56 | -0.61 | -0.78 | 0.17 | -0.71 | -0.89 | 0.18 | -0.49 | -0.63 | 0.14 |
| Phe57/Leu60 | -1.12 | -0.77 | -0.35 | -1.38 | -0.99 | -0.39 | -1.08 | -0.67 | -0.41 |
| Asn59/Thr62 | -0.1 | -0.16 | 0.06 | 0.01 | 0.01 | 0 | -0.03 | 0 | -0.03 |
| Glu61/Gln64 | 0.16 | 0.02 | 0.14 | 0.15 | 0.02 | 0.13 | 0.15 | 0.02 | 0.13 |
| Ile62/Phe65 | -0.02 | 0.02 | -0.04 | -0.03 | -0.01 | -0.02 | -0.01 | 0.01 | -0.02 |
| Val73/Thr76 | -0.04 | -0.02 | -0.02 | -0.01 | -0.01 | 0 | -0.04 | -0.02 | -0.02 |
| Asp77/Gly80 | 0.23 | 0.4 | -0.17 | 0.28 | 0.11 | 0.17 | -0.21 | -0.02 | -0.19 |
| Ser101/Glu104 | -0.01 | 0.1 | -0.11 | 0.01 | 0.11 | -0.1 | -0.01 | 0.11 | -0.12 |
| Lys120/Asn123 | -0.08 | 0.01 | -0.09 | -0.07 | -0.01 | -0.06 | -0.08 | 0.01 | -0.09 |
| Ser124/Cys127 | -0.03 | -0.22 | 0.19 | -0.04 | -0.15 | 0.11 | 0.02 | -0.08 | 0.1 |
| Val127/Ile130 | -0.02 | -0.02 | 0 | -0.02 | -0.02 | 0 | -0.02 | -0.02 | 0 |

[a]All values are in kcal/mol.

[b]$\Delta\Delta E = \Delta E^{FABP4} - \Delta E^{FABP5}$.

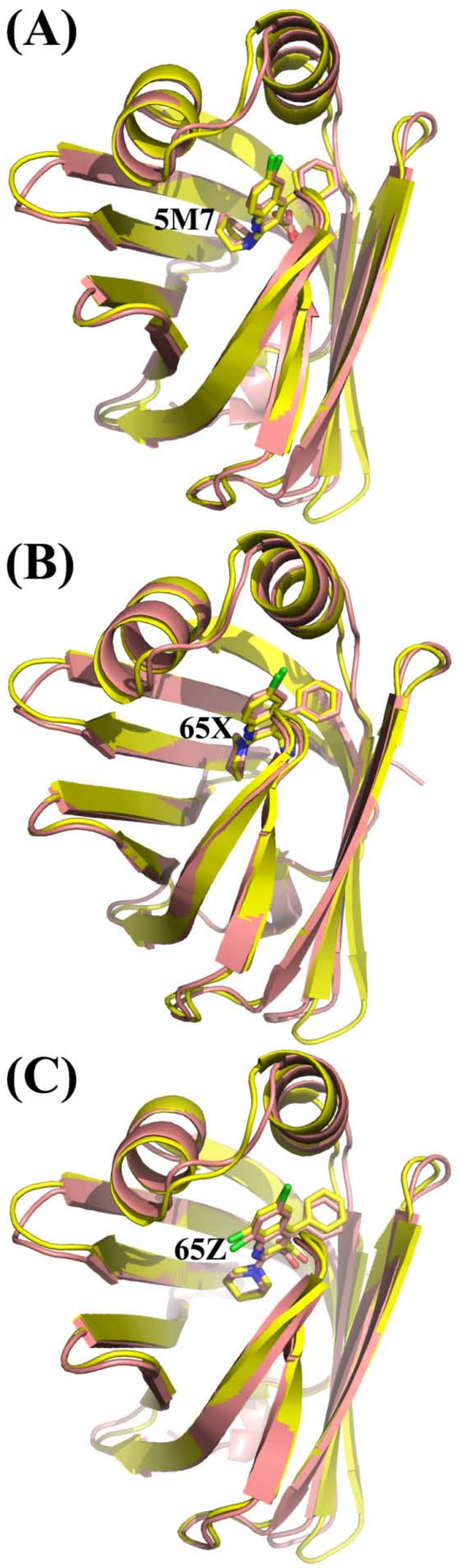


**Figure S1.** Superposition of conformations used in the experimental studies between inhibitor-FABP4 (yellow) and inhibitor-FABP5 (pink) complexes. **(A)** 5M7-FABP4/FABP5, **(B)** 65X-FABP4/FABP5 and **(C)** 65Z-FABP4/FABP5.

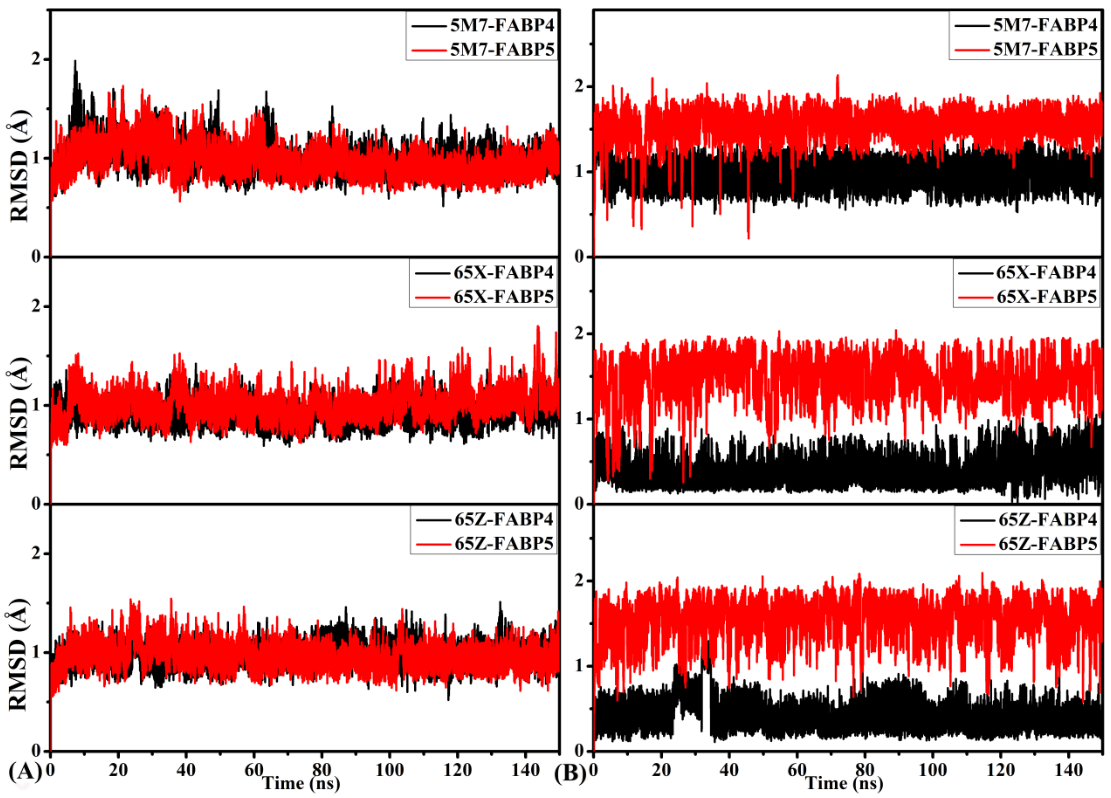


**Figure S2.** Root-mean-square-deviations (RMSDs) of the backbone atoms in FABP4/FABP5 **(A)** and three inhibitors **(B)** relative to their starting structures during the MD simulation as function as time.

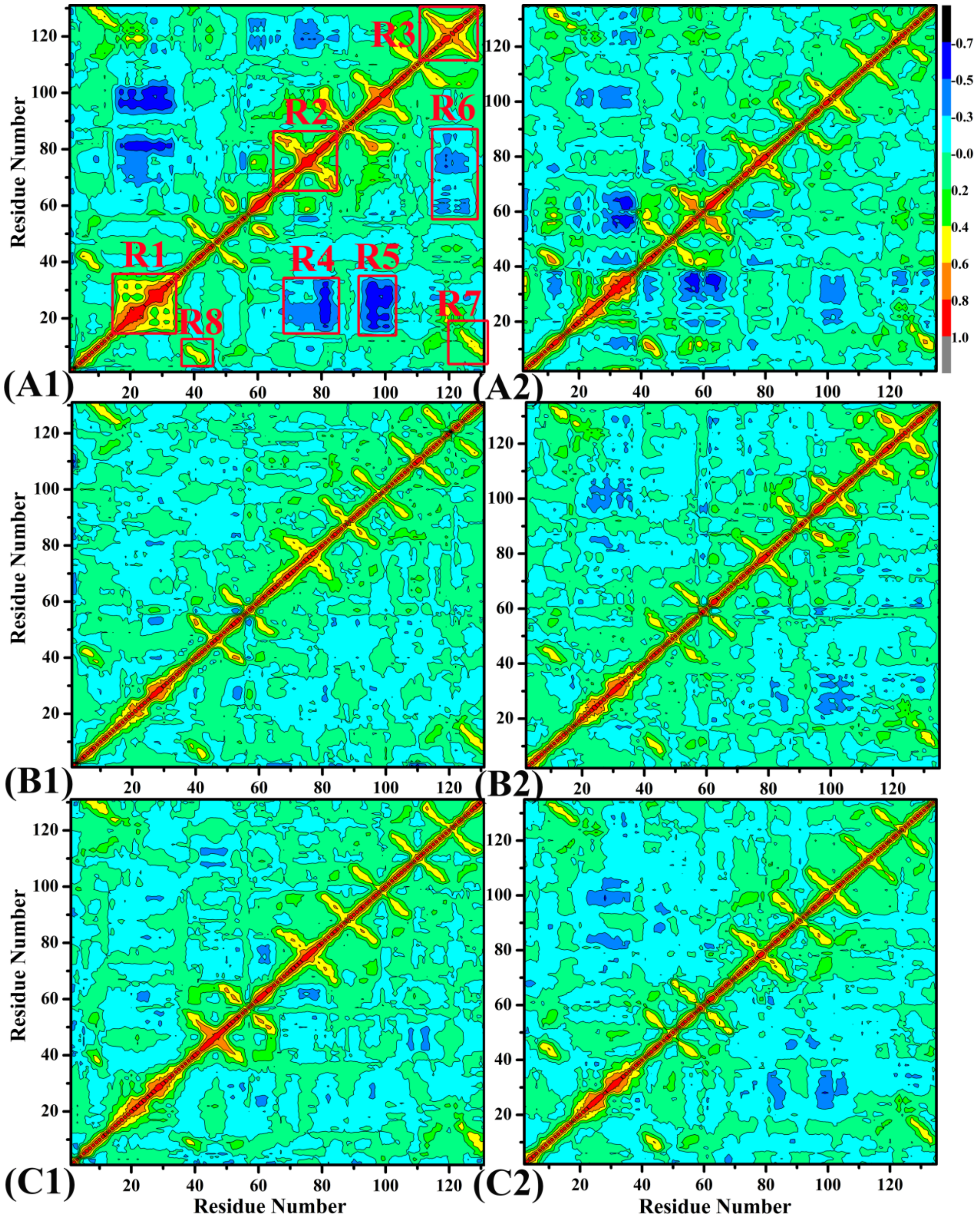


**Figure S3.** Cross-correlation matrices of fluctuations of C$\alpha$ atoms relative to their average positions in six systems: **(A1/A2)** 5M7-FABP4/FABP5, **(B1/B2)** 65X-FABP4/FABP5 and **(C1/C2)** 65Z-FABP4/FABP5.

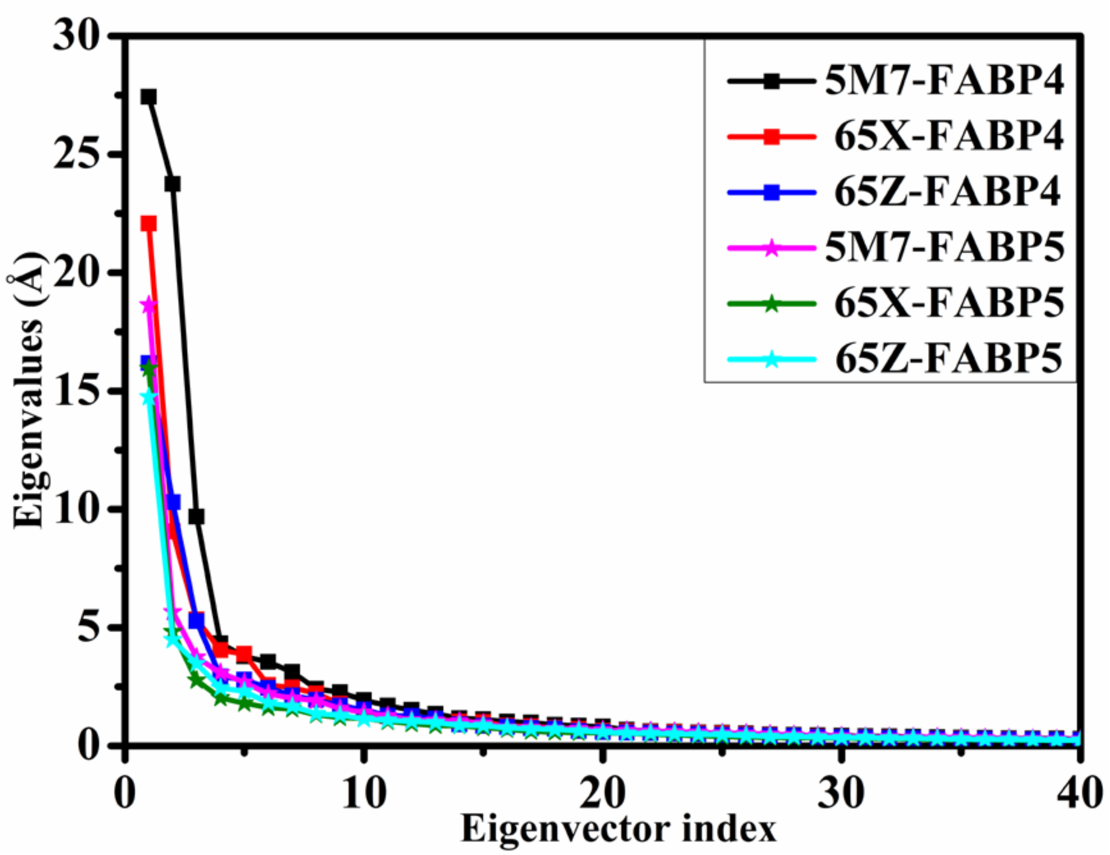


**Figure S4.** Comparison of the eigenvalues plotted against the corresponding eigenvector indices originated from the diagonalization of covariance matrix of C$\alpha$ atoms.
